# Inverse Identification and Design of Thermal Parameters of Woven Composites through a Particle Swarm Optimization Method

**DOI:** 10.3390/ma16051953

**Published:** 2023-02-27

**Authors:** Fei Guo, Xiaoyu Zhao, Wenqiong Tu, Cheng Liu, Beibei Li, Jinrui Ye

**Affiliations:** 1 School of Mechanical and Automotive Engineering, Shanghai University of Engineering Science, Shanghai 201620, China; 2 School of Automotive and Traffic Engineering, Jiangsu University, Zhenjiang 212013, China; 3 Department of Civil Engineering, Zhejiang College of Construction, Hangzhou 311231, China; 4 School of Aerospace Engineering, Beijing Institute of Technology, Beijing 100081, China

**Keywords:** plain woven composites, inverse, optimization design, particle swarm optimization

## Abstract

Designing thermal conductivity efficiently is one of the most important study fields for taking the advantages of woven composites. This paper presents an inverse method for the thermal conductivity design of woven composite materials. Based on the multi-scale structure characteristics of woven composites, a multi-scale model of inversing heat conduction coefficient of fibers is established, including a macroscale composite model, mesoscale fiber yarn model, microscale fiber and matrix model. In order to improve computational efficiency, the particle swarm optimization (PSO) algorithm and locally exact homogenization theory (LEHT) are utilized. LEHT is an efficient analytical method for heat conduction analysis. It does not require meshing and preprocessing but obtains analytical expressions of internal temperature and heat flow of materials by solving heat differential equations and combined with Fourier’s formula, relevant thermal conductivity parameters can be obtained. The proposed method is based on the idea of optimum design ideology of material parameters from top to bottom. The optimized parameters of components need to be designed hierarchically, including: (1) combing theoretical model with the particle swarm optimization algorithm at the macroscale to inverse parameters of yarn; (2) combining LEHT with the particle swarm optimization algorithm at the mesoscale to inverse original fiber parameters. To identify the validation of the proposed method, the present results are compared with given definite value, which can be seen that they have a good agreement with errors less than 1%. The proposed optimization method could effectively design thermal conductivity parameters and volume fraction for all components of woven composites.

## 1. Introduction

Woven composites have excellent structural and thermal properties due to their complex structural characteristics, which have improved interlaminar properties by interweaving fiber bundles. Moreover, functionally graded materials (FGMs) are the new generation of composite materials which overcome the issue of thermal resistance and also delamination in multilayer structures. Delouei et al. [1,2] proposed an analytical solution for the steady-state thermal conductivity of FGMs. However, with the improvement of comfort requirements, in addition to the requirements of interlayer performance in practical application, some materials also have higher requirements for heat conductivity. For example, in order to prevent overheating in the battery working process, the automobile industry has high requirements on the heat dissipation performance of the battery box; in order to maintain the appropriate temperature in the car and improve occupant comfort, the heat insulation performance of the body cladding is highly required. In order to ensure the comfort of residents, the building industry also has high requirements on the thermal insulation performance of materials. For materials with different heat conduction requirements, it is necessary to design each component according to the target heat conduction performance. Furthermore, woven composites have been also used in some thermal protection materials. For example, NASA has developed a kind of woven composite material thermal protection system for extreme environments, where the material’s thermal conductivity plays a key role [3].

Because woven composites are composed of interwoven braided structure and matrix, and braided structure is composed of yarn and matrix, the research on the thermal conductivity of woven composites needs to be carried out at different scales. Fourier’s law is an effective method to study thermal conductivity of homogeneous materials. However, woven composite material is anisotropic material. Therefore, homogenizing is necessary at different scales for thermal conductivity of woven composites. In order to study the heat transfer coefficient using Fourier’s law, homogenization is required at each scale. Some scholars have established multi-scale research models for composites to study the heat conductivity from bottom to top [4,5,6,7,8]. Dasgupta et al. [9] predicted thermal conductivity by analogy between thermal resistance and electrical resistance. They obtained the thermal conductivity of woven composite materials by using the similarity between them. Ning and Seo [10,11] also used the thermoelectric analogy method to study the thermal conductivity of woven composites and proposed a thermal conductivity prediction model. Zhao et al. [12,13] established a progressive homogenization model for plain-woven composites, and extended LEHT to study the influence of internal defects and interface effects on heat conduction of woven composites.

Although the structural characteristics of woven composites can bring excellent material properties, the complexity of the structure also brings some challenges to the design of the materials. Some experimental results have shown research on the thermal conduction performance is necessary for fully exploiting the advantages of composites [14,15]. Kim et al. [16] solved the original thermal conductivity of woven composites by using the superheated electrical analogy model. Genetic algorithm was adopted to optimize the thermal conductivity of woven composites by improving the structural parameters of the materials. Based on the experimental data in the literature [17], researchers determined the thermal conduction coefficient of a specific insulating material with a density of 110 kg/m^3^ and a porosity of 0.94 by numerical simulation according to the Fourier law and deduced the thermal conductivity of nanoparticles by using particle swarm optimization (PSO) method. Da et al. [18] obtained the effective thermal conductivity of micro-three-phase or above materials through the homogenization theory, adopted the multiphase interpolation method to deal with the micro-scale multi-material topology optimization problem, integrated the two-scale optimization problem with the classical homogeneous method, and solved the problem by using the bidirectional evolutionary structure optimization method. Su et al. [19] studied thermal conductivity of TIMs by the finite element method and random thermal network model (RTNM), and combining with genetic algorithm (GA), they established a new set of RTNM program. The proposed method can be used in the case of a given filling load. Finally, they found a multi-scale particle matching method to obtain the maximum effective thermal conductivity. Zhou et al. [20] acquired the sensitivity of the thermal conductivity of woven composites to towards the binding geometric dimensions based on the existing theoretical model formula, and optimized thermal conductivity combined with a gradient-based method, and studied the fiber layout and yarn structure design. On the basis of the existing homogenization analysis and gradient optimization, a complete relationship between the geometrical dimensions of woven reinforcement and the effective thermal conductivity of woven composites is established. Bakar et al. [21] studied the elastic properties of woven composites by combining various genetic optimization algorithms to predict the elastic properties of fabric composites based on the geometric properties and elastic properties of single cells. To obtain the best elastic performances, they recombined parameters of components, including the thickness and volume fraction of yarn, volume fraction of fiber in the yarn, etc. Cherif et al. [22] proposed an optimal method for the structure of plain-woven reinforced composite materials. Through the design of structural parameters of the reinforced materials, such as matrix, yarn diameter, filling rate, etc., the objective function including mechanical and adsorption properties was constructed based on the principles of high filling rate of the blended fabric, small yarn shrinkage and small fiber diameter. Chen et al. [23] researched the influence of different internal phase parameters of unidirectional fiber composites by the optimization method based on homogenization inversing. Zeng et al. [24] simplified the optimization problem of 3D woven composites to a mathematical model problem using a mixed integer to represent the real and integral variables of the 3D fiber structure. They adopted FEM based on represent unit cell to evaluate the objective function, which was carried out in the braided composite modeling software TexGen(v3.12.0, University of Nottingham, Nottingham, UK) and finite element analysis software ABAQUS 2020. Tao et al. [25] researched optimization and lightweight design of three-dimensional woven composite automobile fenders based on PSO, which is combined with Kriging surrogate model to find the optimal combination of component design variables at different scales.

Most existing research focused on the bottom-up prediction of effective responses of woven composites from known constituents’ properties. However, it is demonstrated that in the experimental measurement that properties of fiber or matrix could be changed due to several factors such as manufacturing defects, residual stresses, etc. In addition, a few microscale parameters, such as fiber’s transverse properties, cannot be easily determined through direct measurement. In addition, accurate parameters cannot be designed according to the thermal conductivity of the target by a bottom-up method, and the component parameters need to be adjusted continuously to achieve the target performance. Therefore, although several papers have already conducted research on the design of woven composites, they require a complex process of parameter adjustment. The wide application of woven composite materials determines that the design and research of them need to be more in-depth. This paper presents a top-down approach to study the thermal conductivity of woven composites. Considering that the fibers and matrix have great influence on the macroscopic properties of woven composites, the meso-scale fiber bundle parameters and micro-scale fiber parameters directly affect the macroscopic thermal conductivity of woven composites. According to the study conducted by Zhao et al. [12,13], it is found that both thermal conductivity and volume fraction of fiber have great influence on the macro heat conductivity. Therefore, in this paper, the heat conduction coefficient and fiber volume fraction of transversely isotropic fibers are retroactively deduced according to the target heat conduction coefficient of woven composites. The macro parameters can be obtained experimentally or numerically, so the cost of trial and error in material design can be greatly reduced by retrofitting the parameters of components with the easily obtained macro parameters. Integrating the methods used in material design in existing studies, it can be seen that PSO avoids the calculation of any explicit function expressions and corresponding gradients, ensuring the stability of the optimization process and making it easy to implement in various engineering scenarios. With respect to the micromechanics techniques, locally exact homogenization theory (LEHT) has proven to be a more efficient method compared to the discretization-based numerical approach [4,5,6], such as finite element approach, finite volume method. The method was initially developed by Pindera and his coworkers for unidirectional composites and has been extensively validated with finite element [5,6] and finite volume technique [4,6]. Very recently, LEHT is combined with finite volume technique to formulate a new hybrid homogenization theory by Pindera and his coworker [26,27]. The hybrid technique greatly facilitates accurate and efficient studies of composites with random fiber distributions. Further, the PSO algorithm has excellent global searching capability, which is a type of evolutionary algorithm [28,29]. The analytical-based LEHT greatly facilitates the marriage of those two approaches due to the explicit expression of homogenized properties. Therefore, combining LEHT with PSO is a worthy noticed novelty, which could design and optimize parameters of constituent accurately and efficiently.

In this paper, for the optimization of plain woven composite, a PSO-based inverse method is presented by combing PSO with the multi-scale method & homogenization method. The remainder of the papers is organized as follows: Section 2 establishes a hierarchical multiscale analysis frame and proposes an optimization method for the woven composite. Section 3 gives a brief introduction to particle swarm optimization. The principle of particle swarm optimization and the ideas used in this paper are elaborated in this part. In Section 4, the heat conduction coefficient and volume fraction of fiber are designed for plain woven composites by the inverse method. Finally, conclusions are summarized in Section 5.

## 2. Optimization Scheme Based on Multi-Scale Framework for the Plain-Woven Composites

### 2.1. Plain-Woven Composites Multi-Scale Framework

**Figure 1:**
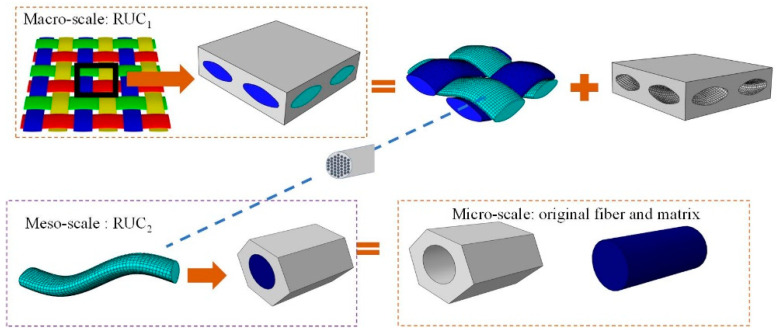
Multi-scale framework of plain-woven composites.

Woven composites are reinforced materials formed by interweaving warp and weft yarn, which have obvious periodical characteristics [30]. From the perspective of composition structure, the multi-scale framework can be represented as Figure 1. The macro-scale woven composite material is composed of woven structure and matrix, meso-scale yarn is composed of fiber and matrix, and microscopic scale is composed of original fiber and matrix. In addition to experimental measurement, the macroscopic thermal conduction coefficients required in practical application can be obtained by analytical model. However, for meso-scale and micro-scale parameters characterization, multi-scale method is necessary. At each scale, the characteristics of the least periodic unit can represent the overall characteristics of the material. Multi-scale frames are common for all woven composites. For this paper, the research object is Carbonized Silica/Phenolic woven composite, whose original components are carbonized in a Silica and phenolic matrix.

### 2.2. Inverse Optimization Method for Woven Composites

**Figure 2:**
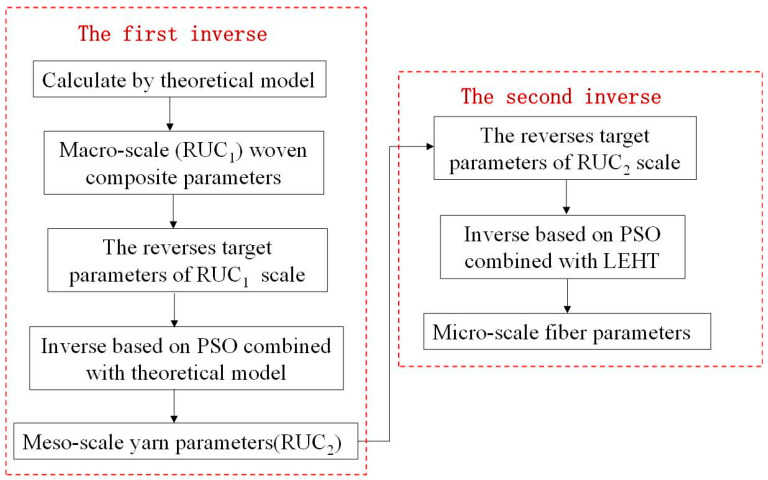
Two-step inverse flowchart for woven composite optimization.

Because of the complex structure of woven composites, the effective thermal conduction coefficients of macroscopic woven composites depend on the thermal conduction coefficients of each scale. There are two research methods: the bottom-up method and top to bottom method. Bottom-up design requires a complicated trial and error process. However, the inverse calculation from top to bottom saves a lot of cost and improves the efficiency of calculation. Several optimization techniques can achieve this goal [30]. However, traditional optimization techniques require the calculation of gradients, which maybe not attainable for complicated real engineering problems. Particle swarm optimization (PSO) avoids these problems [31]. Therefore, the combination of PSO algorithm and inverse method is an effective method for optimal design of woven composites. Similar to the prediction process of thermal conduction coefficient of woven composites, the optimization design process also needs to be carried out step by step. Figure 2 shows the flowchart for the multi-scale composition framework of woven composite material. There are two inverse process to obtain original fiber parameters. Firstly, the theoretical model is introduced into the particle swarm optimization algorithm on the macroscale (RUC_1_). There are some known theoretical models for researching the thermal conduction coefficient of woven composites, which have been given in the Appendix A. As for the inverse of meso-scale, LEHT can calculate the heat conduction coefficient of yarn efficiently and accurately. Then, in terms of the combination of particle swarm optimization algorithm with LEHT theory on the meso-scale (RUC_2_), original fiber parameters can be deduced.

The inverse research includes two inverse processes. The first one is to calculate the effective thermal conduction coefficients of plain-woven composites and backout the thermal conduction coefficients of yarn based on the marriage between theorical model and PSO. The second inverse is to deduce the original fiber thermal conduction coefficients based on the inverse values from the first inverse step.

## 3. Inverse Method Based on PSO

We employed the particle swarm optimization (PSO), first proposed by Kennedy and Eberhart [28,31], which is a gradient-free technique based on simulating the social interactions of birds. To complete the inverse calculation, we first distribute a set of populations (id = 1, 2, …, N). The position of the *i*th particle is X_id_ = (x_1_, x_2_, …, x_D_) _id_, where the parameters are the variables to be optimized. PSO has been used in many engineering fields because of its simple operation and fast convergence.

**Figure 3:**
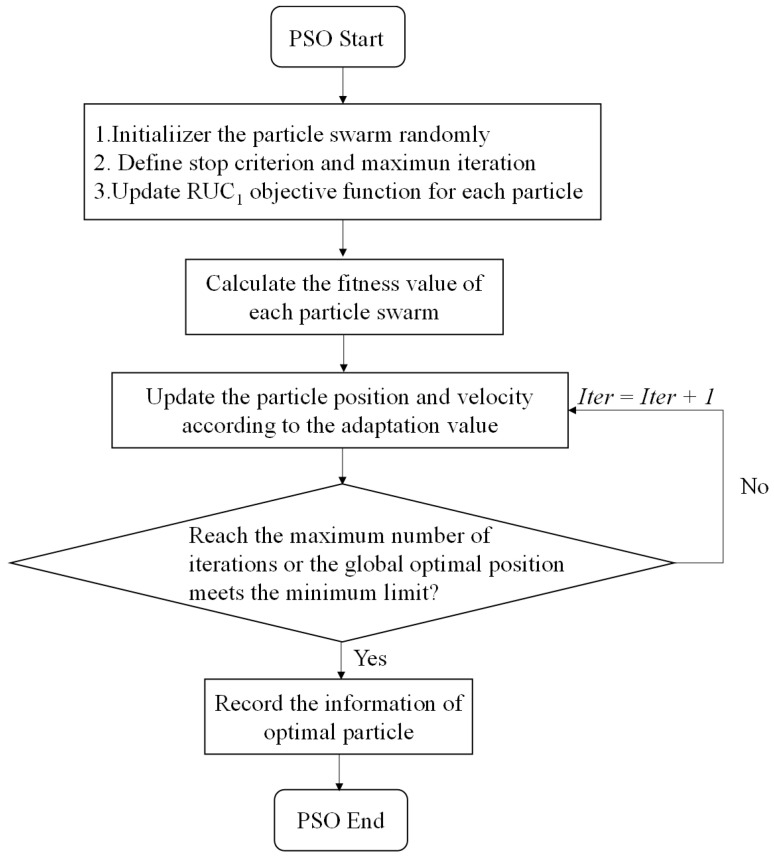
Flowchart of particle swarm optimization algorithm.

The PSO algorithm is inspired by the social behavior pattern of birds. This algorithm is based on the idea that the intelligence of each individual in a population will affect the behavior of the whole flock, which can be visually described as: there is only one piece of food in the specific area (the value range of the target design variable), the goal of the flock is to find out food source. In the whole searching process, each individual passes their own information to each other to let other particles know their position. They can judge whether the optimal solution is found or not by obtained information from others. At the same time, the information of the optimal solution is transmitted to the whole flock. Finally, all the particles can gather around the food source. It turns the problem of finding the optimal solution into the problem of finding a food source iteratively by a group of particles carrying speed and direction in space. At each iteration, every particle will reassess its position and move towards a global goal. This process continues until the maximum iteration is reached. PSO has been widely applied in the engineering field due to its simple implementation and fast searching speed [30]. The basic process is as follows: Initializing the particle position and velocity randomly, and evaluating the fitness value of each particle. The speed and direction of each particle are constantly updated through the cyclic iteration, and the extreme value of the individual and the flock are updated according to the fitness value of the new particle. Figure 3 shows the implementation process of the particle swarm optimization algorithm.

In the standard PSO algorithm, the direction and velocity of particles are updated and iterated by the following equations. In PSO, Equation (1) is used to generate a new velocity according to its present velocity. The new position is associated with the distance between its current position and its own historical best position and the best position of the whole flock. The particle is then moved to a new position according to Equation (2). The maximum number of iterations can be designed in advance as termination criterion:

(1) $$v_{k+1}^{i}=\omega v_{k}^{i}+c_{1}r_{1}(p_{k}^{i}-x_{k}^{i})+c_{2}r_{2}(p_{k}^{g}-x_{k}^{i})$$

(2) $$x_{k+1}^{i}=x_{k}^{i}+v_{k+1}^{i}$$

where $x_{k}^{i}$ and $v_{k}^{i}$ represents the position and velocity of the particle i in the *k*th iteration, respectively. $p_{k}^{i}$ represents the optimal position found by the *k*th particle, $p_{k}^{g}$ represents the global optimal position found by the whole particle flock, and the particles move iteratively towards $p_{k}^{i}$ and $p_{k}^{g}$. $c_{1}$ and $c_{2}$ are learning factors. $r_{1}$ and $r_{2}$ are random number between [0, 1]. ω is a relevant factor used to balance the globally and locally optimal solutions. When its value is large, the global optimization ability is stronger than the local optimization ability. On the contrary, when its value is small, global optimization ability is weaker than local optimization ability. The introduction of ω, particle swarm optimization greatly improves the computational efficiency of particle swarm optimization algorithm. For different problems, it can be adjusted according to the needs of the global and local search ability [30], defined as:

(3) $$\omega (iter)=\frac{iter_{\max}-iter}{iter_{\max}}\times (\omega_{ini}-\omega_{gnd})+\omega_{gnd}$$

where iter is the current iteration, $iter_{\max}$ is the max iteration, $\omega_{ini}$ is initial inertia weight, and $\omega_{gnd}$ is the inertia factor when iterating to the maximum evolutionary algebra. The influence of particle’s own experience information and other particle’s experience information on particle trajectory is determined by learning factors and reflects the information exchange among particle groups. If the value $c_{1}$ is set too large, the particle will wander locally. Accordingly, if the value of $c_{2}$ is set too large, the particle will converge prematurely before the global optimal solution is found. In this study, to obtain the value of optimal variables of plain woven composites, the objective function can be defined by minimizing the difference between the results generated by the meso-optimization results $f^{\mathrm{PSO}}(X)$ and the target value or experimental results $f^{T\arg\mathrm{et}}(X)$. The objective function can be expressed as:

(4) $$\mathrm{Obj}=\min\frac{\sum_{k=1}^{N}\left|f^{\mathrm{PSO}}(X)-f^{\mathrm{Target}}(X)\right|}{\sum_{k=1}^{N}\left|f^{\mathrm{Target}}(X)\right|}$$

For all particles carrying different information, “min” means minimizing the target function. The number of iterations needs to be met by constantly updating the particle X carrying optimization parameters until the termination condition is met or the maximum number of iterations is reached. In practical applications, many optimization problems will be encountered, and these optimization problems are generally multi-objective. Taking the optimization problem of thermal conductivity of composite materials as an example, since the research object is anisotropic composite materials, it is necessary to balance the transverse thermal conductivity and axial thermal conductivity to make the best choice. This chapter takes the minimum value problem as an example to carry out the multi-objective optimization.

## 4. Inverse and Optimization of the Plain-Woven Composites

In order to validate the effectiveness of proposed inverse method, this chapter firstly compares the inverse values with the original values in existing literatures [32], including the axial and transverse heat conduction coefficients of original fibers. Because the effective thermal conduction coefficients of woven composites are closely related to the heat conductivity performance and volume fraction of fiber, the design and optimization of fiber performance parameters are also necessary.

### 4.1. Inverse Identification on the Thermal Conduction Coefficients of Woven Composite Original Fiber

Based on the structural hierarchical characteristics of woven composites, the inverse calculation of its thermal conductivity is also required, which is, respectively, the inverse calculation from macroscopic thermal conductivity to yarn scale thermal conductivity and the inverse calculation from yarn scale thermal conductivity to microscopic thermal conductivity.

Based on the macroscopic parameters of Carbonized Silica/Phenolic woven composites studied in former literature, this section used particle swarm optimization algorithm to reverse the thermal conduction coefficient of transverse isotropic fibers. The agreement between the theoretical formula and the existing model has been verified in a former study. In order to find the appropriate parameters of original fiber and matrix, the theoretical model is used to reverse the yarn parameters, and then the inverse values are substituted into the LEHT model for the inverse of the fiber and matrix microscopic thermal conductivity. For the inverse of the original thermal conduction coefficients of the fiber, the difference between the existing calculation of the thermal conduction coefficients and the one calculated by the optimized value is minimized as the objective function. The number of iterations in the calculation of this section is 20. Here, the reverse derivation of the fiber and matrix thermal conduction coefficients are performed using the fiber volume fraction in the yarn as 0.6. The selected design variables are: $X=\begin{array}{cc} {[x}_{1} & x_{2} \end{array}{]}^{T}={\left[\begin{array}{cc} k_{ya} & k_{yt} \end{array}\right]}^{T}$, where $k_{ya}$ and $k_{yt}$ represent axial and transversal thermal conductivity, respectively. $k_{m}$ is thermal conductivity of matrix and $v_{y}$ is volume fraction of yarn. The objective function when inversing the thermal conductivity of the yarn (corresponding to the first inverse in Figure 2) is:

(5) $$\begin{array}{l} \mathrm{func}\_\mathrm{opt}=\min f\{k_{ya},k_{yt}\}=\min\{\mathrm{abs}[(0.5*(v_{y}*k_{ya}+(1-v_{y})*k_{m})+\\ 0.5*k_{m}*[((1-v_{y})*k_{m}+(1+v_{y})*k_{yt})/((1-v_{y})*k_{yt}+(1+v_{y})*k_{m})])-k_{\mathrm{present}}]\} \end{array}$$

which theorical model comes from Parallel Model and Pilling Model in reference [25]:

(6) k=12(kParallel+kPilling)

The accuracy of the thermal conductivity of yarn used by LEHT theory has been verified in former study [12,13], and the accuracy of the proposed method is another important consideration when inversing the thermal conduction coefficients of original fiber based on the back-projected thermal conductivity of yarn. Here, we substitute the final optimal parameters into LEHT to generate the effective axial and transverse thermal conductivity of the fiber at different volume fractions. Further, we embed the procedure of LEHT (detail can be seen in reference [31,33,34,35]) into the PSO program by:

(7) $$\left[k_{11,}k_{22}]=\mathrm{Homogenization}\_\mathrm{Conductivity}\_\mathrm{Interphase}(k_{11,}k_{22}\right)$$

where $k_{11}$ and $k_{22}$ is axial and transverse thermal conductivity, respectively.

The objective function corresponding to the second inverse in Figure 2 is:

(8) $$\mathrm{func}\_\mathrm{opt}=\min f\{k_{11,}k_{22}\}=\min\{\mathrm{abs}(k_{22}-k_{yt})/k_{yt}+\mathrm{abs}(k_{11}-k_{ya})/k_{ya}\}$$

**Figure 4:**
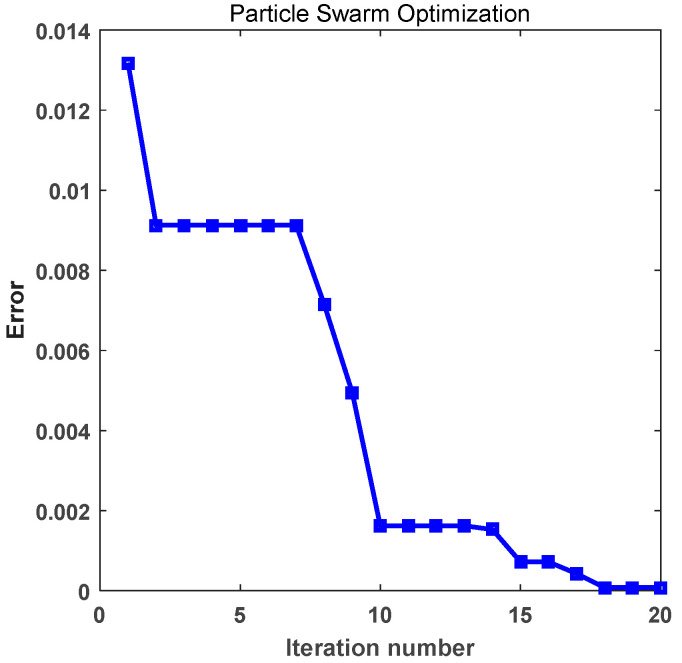
Calculation error of particle swarm optimization.

Through the particle swarm optimization algorithm, Table 1 shows inverse value and calculation error. The inversed axial and transverse thermal conduction coefficient by the particle swarm algorithm is 0.725638 W/(mK) and 0.382584 W/(mK), respectively, and the original fiber axial and transverse thermal conductivities in the literature [32] are 0.726 W/(mK) and 0.383 W/(mK).Therefore, the inverse thermal conductivity results of the fiber deduced by the particle swarm optimization algorithm are reliable. Figure 4 shows the calculation error of different iteration times. At the beginning of the iteration, the information carried by each particle is relatively simple, which is far away from the target value, resulting in a large error. With the progress of iteration, particles in the flock constantly exchange information with each other. Each particle obtains more and more information about the target value, and moves nearer and nearer to the global optimal solution, and the error decreases gradually. When the maximum number of iterations is reached, all particles have almost moved to the target value, that is, the global optimal solution is found. It is clear that by the time the global optimal solution is found, the error is already very small.

### 4.2. Optimization Example of Plain-Woven Composites

In the process of material design, people hope to give full play to the excellent performance of various components, so the design process is imperceptibly infiltrated into the idea of optimization, and according to the target performance of the property parameters design actually uses the idea of reverse inference. Section 4.1 has verified the effectiveness of using a particle swarm optimization algorithm to study the thermal conductivity of woven composites. However, in the actual use of materials, the components of composite materials are often determined according to the required macro parameters. In the case of given macro parameters, determining reasonable volume fraction of fiber and thermal conductivities of fiber will be the key to improve the utilization rate of fiber. Herein, we gave an optimization example by utilizing the proposed method.

**Figure 5:**
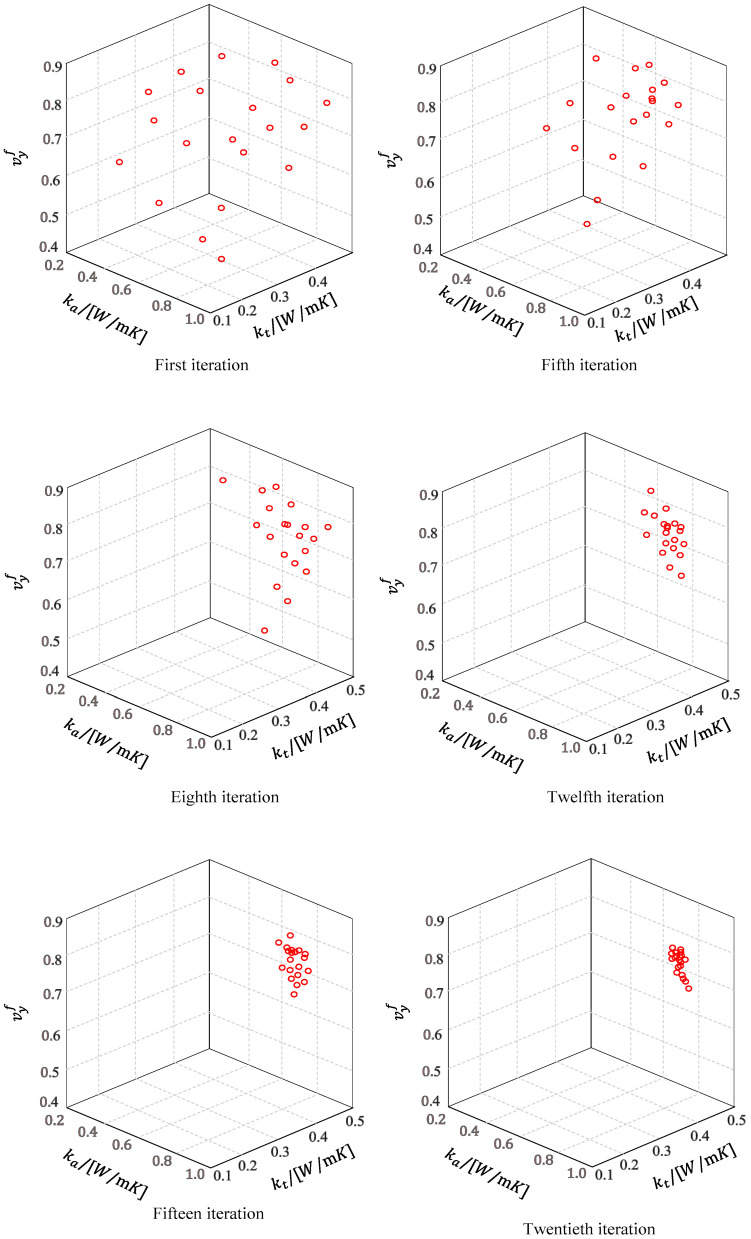
Particle positions of different iterations.

As for the macroscopic thermal conductivity of composite materials, it is affected by many factors. The thermal conductivity parameters of fiber play a key role. According to the research results of Carbonized Silica/Phenolic macroscopic thermal conductivity in the reference [32], the fiber volume fraction and thermal conductivity were designed reasonably. The objectively macroscopical axial and transverse thermal conductivity is 2.5 W/(mK) and 1.7 W/(mK), respectively. Firstly, thermal conductivity coefficients of yarn can be obtained by the first inverse. The axial and transverse thermal conductivity coefficient is 2.4125 W/(mK) and 1.7726 W/(mK). Then, combined with LEHT, original fiber thermal conductivity and fiber volume fraction can be inversed. Table 2 listed optimized value of different variables. Figure 5 shows the positions of particles in different iterations. It can be found that when the 20th iteration is carried out, particles have tended to the same target value, and then the algorithm tends to converge.

## 5. Conclusions

In order to design the target thermal conductivity of plain-woven composites, the present work proposed to employ an analytical model and LEHT combined with particle swarm optimization algorithm at a mesoscale and microscale based on the hierarchical and periodic characteristic of woven composites. It should be noticed that LEHT is efficient and accurate in calculating the thermal conductivity of yarn and easy to combine with particle swarm optimization algorithm. Additionally, the quantitative calculation of thermal conductivity is carried out by combining multiscale and homogenization methods. The main conclusions are as follows:

(1) The inverse method can greatly reduce the design cost of woven composites, and the performance advantage of the constituent materials can be fully exploited by top to bottom method.
(2) The PSO-based inverse method can calculate the homogenized thermal properties of woven composites at different scales and the thermal conduction coefficients at different scales can be backed out sequentially. In order to verify the feasibility of the proposed method, the thermal conduction coefficients of Carbonized Silica/Phenolic woven composite fiber were inversed, and fiber’s thermal conduction coefficients were compared with that of the original fiber. The comparison results showed that the inverse values were in good agreement with the testing value.
(3) LEHT has been formed as a packaged program. When combining LEHT with PSO algorithm, researchers just require minimal effort in constructing input data file to execute the computer program, which can be efficiently used by researchers with little thermotic exposure.

## Figures and Tables

**Table 1:** Design variables and design scope.

Design Variable	Variable Description	Design Scope	Original Value [25]	Inverse Value	Error
$x_{1}$	$k_{a}$	[0.5, 1]	0.726 W/(mK)	0.725638 W/(mK)	0.055%
$x_{2}$	$k_{t}$	[0, 0.5]	0.383 W/(mK)	0.382584 W/(mK)	1.3%

**Table 2:** Optimized design parameters of Carbonized Silica/Phenolic woven composites.

Design Variable	Variable Description	Design Scope	Design Value
$x_{1}$	$k_{a}$/[W/(mK)]	[0.5, 1.0]	0.785
$x_{2}$	$k_{t}$/[W/(mK)]	[0.2, 0.5]	0.342
$x_{3}$	$V_{y}^{f}$	[0.5, 0.8]	0.763

$k_{a}$ represents axial thermal conductivity of fiber, $k_{t}$ represents transverse thermal conductivity of fiber, and $V_{y}^{f}$ represent fiber volume in the yarn.

## Data Availability

All data, models, or code that support the findings of this study are available from the corresponding author upon reasonable request.

## Appendix A

**Table A1:** Several calculation equations of theorical model.

Model	Equation
Parallel model	$k_{pa}=v_{f}k_{f}+(1-v_{f})k_{m}$
Series model	$k_{s}=\frac{1}{v_{f}/k_{{}_{f}}+(1-v_{f})k_{m}}$
Clayton model	$k_{c}=\frac{k_{m}}{4}{\left[\sqrt{{\left(1-v_{f}\right)}^{2}{\left(\frac{k_{{}_{f}}}{k_{m}}-1\right)}^{2}+4\frac{k_{{}_{f}}}{k_{m}}}-(1-v_{f})(\frac{k_{{}_{f}}}{k_{m}}-1)\right]}^{{}^{2}}$
Pilling model	$k_{p}=k_{m}\frac{(1-v_{f})k_{m}+(1+v_{f})k_{{}_{f}}}{(1-v_{f})k_{{}_{f}}+(1+v_{f})k_{m}}$
Russell model	$k_{r}=k_{m}\frac{v_{f}^{2/3}+\frac{k_{m}}{k_{f}}(1-v_{f}^{2/3})}{v_{f}^{2/3}-v_{f}+\frac{k_{m}}{k_{f}}(1+v_{f}-v_{f}^{2/3})}$
Maxwell model	$k_{\max\mathrm{well}}=k_{m}\frac{k_{f}+2k_{m}+2v_{f}(k_{f}-k_{m})}{k_{f}+2k_{m}-v_{f}(k_{f}-k_{m})}$
